# Glucocorticoid Withdrawal—An Overview on When and How to Diagnose Adrenal Insufficiency in Clinical Practice

**DOI:** 10.3390/diagnostics11040728

**Published:** 2021-04-20

**Authors:** Katarzyna Pelewicz, Piotr Miśkiewicz

**Affiliations:** Department of Internal Medicine and Endocrinology, Medical University of Warsaw, 02-091 Warsaw, Poland; katarzyna.pelewicz@gmail.com

**Keywords:** adrenal insufficiency, cortisol, glucocorticoid treatment, glucocorticoid withdrawal, glucocorticoid-induced adrenal insufficiency

## Abstract

Glucocorticoids (GCs) are widely used due to their anti-inflammatory and immunosuppressive effects. As many as 1–3% of the population are currently on GC treatment. Prolonged therapy with GCs is associated with an increased risk of GC-induced adrenal insufficiency (AI). AI is a rare and often underdiagnosed clinical condition characterized by deficient GC production by the adrenal cortex. AI can be life-threatening; therefore, it is essential to know how to diagnose and treat this disorder. Not only oral but also inhalation, topical, nasal, intra-articular and intravenous administration of GCs may lead to adrenal suppression. Moreover, recent studies have proven that short-term (<4 weeks), as well as low-dose (<5 mg prednisone equivalent per day) GC treatment can also suppress the hypothalamic–pituitary–adrenal axis. Chronic therapy with GCs is the most common cause of AI. GC-induced AI remains challenging for clinicians in everyday patient care. Properly conducted GC withdrawal is crucial in preventing GC-induced AI; however, adrenal suppression may occur despite following recommended GC tapering regimens. A suspicion of GC-induced AI requires careful diagnostic workup and prompt introduction of a GC replacement treatment. The present review provides a summary of current knowledge on the management of GC-induced AI, including diagnostic methods, treatment schedules, and GC withdrawal regimens in adults.

## 1. Introduction

Adrenal glands are composed of the adrenal medulla and adrenal cortex. The medulla is responsible for the secretion of adrenaline, noradrenaline, and dopamine. The adrenal cortex consists of three layers: zona glomerulosa, fasciculata, and reticularis, which produce mineralocorticoids (aldosterone), glucocorticoids (GCs) (11-deoxycorticosterone, corticosterone, and cortisol), and androgens (mostly dehydroepiandrosterone (DHEA), DHEA sulfate (DHEA-S), and androstenedione), respectively. Cortisol secretion depends on the proper function of the hypothalamic–pituitary–adrenal (HPA) axis. Hypothalamic corticotropin-releasing hormone (CRH) stimulates the secretion and synthesis of the adrenocorticotropic hormone (ACTH) in the anterior pituitary. ACTH triggers the release of cortisol and adrenal androgens by binding to the melanocortin-2 receptor on the cells of the zona fasciculata and zona reticularis of the adrenal cortex [1].

Adrenal insufficiency (AI) is a condition resulting from the deficit of GCs. In primary AI, decreased cortisol secretion is caused by altered function of the adrenal glands and is accompanied by the lack of mineralocorticoids and adrenal androgens as well [2]. The reason for secondary AI is impaired secretion of ACTH, either due to pituitary conditions or due to decreased hypothalamic secretion of CRH. It is most commonly caused by chronic therapy with GCs (GC-induced AI) [2]. The symptoms of secondary AI result from lack of GCs only, as mineralocorticoid secretion is usually preserved, being dependent on the renin-angiotensin system.

A variety of GC preparations are used for the treatment of inflammatory, autoimmune disorders, and malignancies. As many as 1–3% of the population are currently on GC treatment [3,4]. Commonly used GCs differ in their anti-inflammatory potency, mineralocorticoid activity, and duration of biological effect, and therefore, ACTH suppression (Table 1). Prednisone, methylprednisolone, and dexamethasone are used in chronic therapy because of a stronger anti-inflammatory activity and lesser sodium retention effect than hydrocortisone [5]. Hydrocortisone is preferably used as a replacement therapy in AI as its short duration of action allows the HPA axis to recover between doses. Exogenous GCs suppress CRH release and therefore ACTH secretion by negative feedback. It primarily leads to an inadequate adrenal response to stress factors and/or a decreased baseline GC levels. A prolonged (more than 4–6 weeks) insufficient ACTH secretion leads to an atrophy of the zona fasciculata and reticularis and decreased ability to secrete cortisol [6]. Tapering the dose of GCs helps the recovery of the HPA axis by resulting in the increase of ACTH level and subsequent restoration of normal adrenal function and cortisol secretion.

It is certain that the administration of oral GCs, long-term and in high doses, may cause GC-induced AI [7,8,9,10]. However, recent studies have found that even short-term (<4 weeks) or low-dose (<5 mg prednisone equivalent per day) GC therapy can alter the function of the HPA axis [11,12]. Abrupt reduction or cessation of exogenous GCs may precipitate symptoms of AI or lead to adrenal crisis [13]. The probability of the suppression of the HPA axis cannot be estimated given the potency, dose, or duration of GC treatment as it does not only depend on these factors but also on different GC metabolism between patients [8,14,15]. Drugs inducing the enzyme CYP3A4 may also affect the metabolism of cortisol, resulting in its faster inactivation to 6β-hydroxycortisol, hence, a need for an increased dose of exogenous GCs per day [16]. Several studies have also proven that inhalation, topical, nasal, intra-articular, as well as intravenous (IV) administration of GCs may cause the suppression of the HPA axis [8,17,18,19,20,21,22,23]. Broersen et al. [7] estimated the percentages of patients diagnosed with AI after GC withdrawal at 48.7% for oral administration, 7.8% for inhalation, 4.7%, and 4.2% for topical and nasal administration, respectively.

Recent studies have shown that the treatment with an IV methylprednisolone administered every week protocol (cumulative dose of 4.5 g) does not cause GC-induced AI [24,25,26,27]. It was, however, proven that this therapy affects adrenal function, causing more severe impairment of DHEA-S secretion than that of cortisol [25]. This subject requires further investigation, as the possibility of the HPA axis suppression in patients receiving IV GCs has not been sufficiently investigated.

## 2. Diagnosing GC-Induced AI

**Figure 1:**
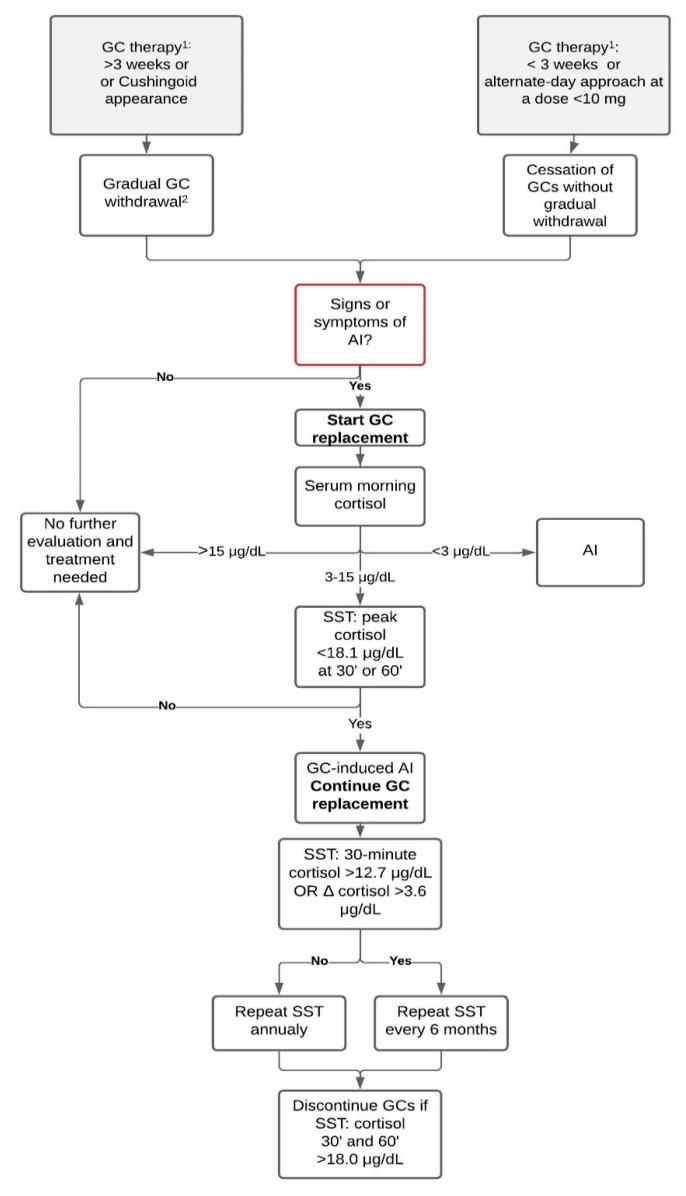
Diagnostic algorithm for patients before and after glucocorticoid withdrawal. Diagnostic workup should never postpone the start of GC replacement treatment when suspecting adrenal crisis. Data from: Furst et al. Glucocorticoid withdrawal. UpToDate. Accessed on 29 March 2021; Fleseriu et al. Hormonal replacement in hypopituitarism in adults: an Endocrine Society Clinical Practice Guideline (2016); Pofi et al. The short synacthen (corticotropin) test can be used to predict recovery of hypothalamo–pituitary–adrenal axis function (2018). Abbreviations: AI, adrenal insufficiency; GC, glucocorticoid; SST, short stimulation test with synthetic ACTH; ^1^ prednisone or equivalent; ^2^ as described in the GC withdrawal paragraph. Δ cortisol: 30-min cortisol minus basal cortisol.

The diagnosis of GC-induced AI in adults is established by detecting low baseline and/or stimulated serum cortisol and requires the etiology of the condition to be determined and introduction of the appropriate replacement treatment (Figure 1).

### 2.1. Symptoms of AI

Symptoms of AI are unspecific and, therefore, can be easily overlooked. Patients may experience fatigue, lack of energy, nausea, vomiting, loss of appetite, weight loss, myalgia, and abdominal pain. The most frequent biochemical finding is hyponatremia, while other electrolyte abnormalities such as hyperkalemia and hypercalcemia are found only in primary AI. In GC-induced AI, patients may present with pale skin as a result of decreased activation of skin melanocortin 1 receptor by ACTH. Characteristic hyperpigmentation of the skin occurs only with primary AI. Clinical suspicion of AI always requires performing diagnostic workup. Diagnosing AI is challenging, especially because missing the diagnosis can potentially be life-threatening.

### 2.2. Baseline Measurements

Baseline serum cortisol can be useful as a screening method for AI. Secretion of cortisol follows a circadian rhythm and peaks between 6:00 and 9:00 a.m. For that reason, a reference range was established based on the samples collected between 8:00 and 9:00 a.m., and therefore, morning serum cortisol should be measured at this time. As cortisol is 90% bound to CBG, states altering CBG levels (e.g., inflammation, pregnancy, critical illness) have to be considered before making a diagnosis of AI [28]. It has been established that serum cortisol values below 3 μg/dL rule in the diagnosis of AI, while levels above 18 μg/dL practically rule out AI [29,30,31]. Lower cutoffs for morning cortisol levels may vary for different immunoassays [32]. Patients with both normal and impaired HPA axis may present with serum cortisol levels ranging from 3 μg/dL to 18 μg/dL. The Endocrine Society suggests performing dynamic testing, which is essential for making the diagnosis of AI, when the morning cortisol level is between 3 and 15 μg/dL [33].

Salivary cortisol level is independent from CBG concentrations and reflects free serum cortisol, which is the active fraction. It is suggested as an alternative for serum cortisol measurement [34,35], especially when CBG levels are altered [36]. Methods for sampling salivary cortisol does not require blood collection, therefore, a stress reaction causing an increase of cortisol is insignificant [37]. However, the use of salivary cortisol in the diagnosis of AI is limited due to a lack of validated reference ranges [38]. Various studies have estimated different cutoff points for morning salivary cortisol and a lower cutoff point has not been yet established [35,37,39,40]. Moreover, the specificity of the assays used for salivary cortisol measurement (first and second-generation electrochemiluminescence assays and liquid chromatography-mass spectrometry) was proven to be low [38].

ACTH level in plasma has a low value in diagnosing GC-induced AI, as its values can be both low and normal in this condition. Detecting clearly elevated ACTH level (>100 pg/mL) may be helpful in ruling out GC-induced AI as it is characteristic of primary AI [41].

DHEA-S level along with baseline serum cortisol level might be helpful in evaluating adrenal function [42]. Normal age- and sex-adjusted serum DHEA-S level predict a proper HPA axis function with a sensitivity of 87.1%, a specificity of 86.7% and a positive predictive value of 93.1% [43]. However, a recent study indicated that DHEA-S alone is a worse parameter for diagnosis of GC-induced AI compared to morning serum cortisol level [11].

### 2.3. Dynamic Tests

It is essential to perform dynamic tests when evaluating adrenal cortisol reserves. Diagnosing GC-induced AI should be performed after tapering the GC dose to the equivalent of ≤5 mg/day of prednisone, preferably hydrocortisone, as long-acting GCs may alter the results of cortisol levels for at least 48 h [33,44]. Hydrocortisone should be discontinued for 24 h before dynamic tests [44,45]. Diagnostic tests for AI include a short stimulation test with synthetic ACTH (SST), insulin tolerance test (ITT), metyrapone stimulation test, CRH stimulation test, and glucagon stimulation test.

SST measures the adrenal response to exogenous synthetic ACTH and is routinely used for the diagnosis of AI. It was judged reliable compared to ITT [46,47] and is safe for the patients. Cortisol level is usually assessed at baseline, 30 and 60 min after IV administration of 250 μg (standard dose test-SDT) or 1 μg (low dose test-LDT) of synthetic ACTH. Because of the low sensitivity of both tests, they are more useful in ruling in secondary AI, more than ruling out the diagnosis [48]. However, a peak cortisol level higher than 18–20 μg/dL (depending on assay-specific reference ranges) practically rule out GC-induced AI [44,47]. Meta-analyses have demonstrated a moderate, yet similar, diagnostic accuracy of standard and low dose stimulation tests in secondary AI [48,49]. While SDT is considered the “gold standard” for establishing primary AI diagnosis [50], some researchers favor LDT in making the diagnosis of secondary AI [51].

ITT measures the response of the whole HPA axis to hypoglycemia, which is one of its most potent stimulants. Patients receive soluble insulin (0.1 U/kg, IV), and the cortisol level is measured 15 min before the insulin injection and every 15 min to 1 h and every 30 min to 2 h thereafter. AI is diagnosed if peak cortisol concentration is <18 μg/dL [52]. ITT is valid when the level of glucose falls below 40 mg/dL and the signs and/or symptoms of hypoglycemia are present. For that reason, ITT has many contraindications (>60 years, cardiovascular or cerebrovascular disease, pregnancy) and potentially dangerous side effects such as neuroglycopenia [44].

Metyrapone, CRH, and glucagon stimulation tests are less frequently performed. They are useful in the assessment of the HPA axis function and may be helpful in diagnosing GC-induced AI. Metyrapone inhibits the conversion from 11-deoxycortisol to cortisol and causes the lowering of cortisol levels. In healthy individuals, it leads to a subsequent rise in ACTH and 11-deoxycortisol level, which is measured in the morning after metyrapone administration 8 h earlier. A lack of increase of 11-deoxycortisol can indicate ACTH deficiency.

CRH stimulation test requires administration of CRH (1 μg/kg IV) and evaluation of cortisol and ACTH levels at baseline and at 15, 30, 45, 60, 90, and 120 min. In those with secondary AI, there is little or no ACTH response. CRH stimulation test is not commonly used because of the high cost of the CRH peptide and low sensitivity for detecting AI [53]. The glucagon test involves evaluation of cortisol level at 90 min and then every 30 min to 4 h after the intramuscular injection of 1 mg glucagon, which stimulates ACTH secretion. Detecting insufficient cortisol response during the test is suggestive of AI. The utility of metyrapone, glucagon, and CRH stimulation tests in diagnosing GC-induced AI has not yet been sufficiently investigated. 

For diagnosis of GC-induced AI the use of LDT is recommended [45]. Some authors suggest that LDT has higher sensitivity than SDT [54,55,56]. It is attributed to the fact that doses of ACTH used during LDT mimic physiological stimulation of the adrenal glands, while the supraphysiological dose used in SDT may be sufficient to produce a response in partially atrophied glands [54]. Two main concerns have been raised regarding LDT use. 1 µg preparation is not commercially available, therefore preparing a 1 µg dose of ACTH requires appropriate diluting a 250- µg vial. A lack of a standardized dilution protocol may lead to the administration of variable doses of ACTH [57]. Another concern was proof that administration of synthetic ACTH via long plastic tubing may lead to false results of LDT due to the adherence of synthetic ACTH to the plastic [58].

### 2.4. MRI of the Hypothalamic-Pituitary Area

In case of a suspicion of GC-induced AI, other causes of central AI should be excluded, and MRI of the hypothalamic–pituitary area should always be considered.

## 3. GC Withdrawal

GC withdrawal usually occurs after reaching the best or optimal therapeutic outcome. Screening for AI is not generally recommended after every GC withdrawal. It is suggested to assess the risk of the HPA axis suppression, which can be classified as probable, intermediate/uncertain, and improbable (Table 2) [45]. Patients with a probable and intermediate/uncertain HPA axis suppression risk require a gradual decrease of GC dose. Abrupt cessation of GCs in these patients can lead to the development of AI symptoms. The patients may also experience withdrawal symptoms that are unspecific and may mimic AI [59].

GCs may be reduced according to the following scheme [45]:

- 5 to 10 mg/day every one to two weeks from an initial dose >40 mg/day of prednisone (or equivalent);
- 5 mg/day every one to two weeks at prednisone doses ranging from 40 to 20 mg/day;
- 2.5 mg/day every two to three weeks at prednisone doses ranging from 20 to 10 mg/day;
- 1 mg/day every two to four weeks at prednisone doses ranging from 10 to 5 mg/day;
- 0.5 mg/day every two to four weeks at prednisone doses <5 mg/day.

Tapering GCs according to the mentioned regimen does not always prevent symptoms of AI. If patients develop signs or symptoms of AI during or after GC withdrawal, then dynamic testing of adrenal function should be performed.

GCs can be discontinued without previous dose tapering in patients with an improbable risk of HPA axis suppression (Table 2). The HPA axis suppression is uncommon with prednisone doses less than 5 mg/day; however, some studies proved that even treatment with physiological doses of GCs might precipitate AI [11,31].

## 4. Recovery of the HPA Axis

There are no specific guidelines concerning the evaluation of the recovery of the HPA axis. Many studies have shown that GC-induced AI may last even up to 2–4 years after GC withdrawal, as in most cases, adrenal atrophy is slowly reversible [8,11,60,61,62]. It is suggested that after 4 years from the diagnosis of GC-induced AI, recovery of the adrenal function is unlikely to occur [63].

Performing SST can also be used to assess the HPA axis recovery after the diagnosis of GC-induced AI [63]. Repeating SST annually is suggested if the patient shows a response to SST with delta cortisol <3.6 μg/dL (where delta cortisol means a cortisol value at 30-min minus basal cortisol value) or 30-min cortisol <12.7 μg/dL. SST should be performed every 6 months if delta cortisol is >3.6 μg/dL or 30-min cortisol >12.7 μg/dL. If the response in SST is adequate, GC replacement can be safely withdrawn.

## 5. Treatment

The treatment of GC-induced AI is a physiological replacement of GCs. Current estimates of endogenous daily cortisol production averages 6–11 mg/m^2^/day [64,65], an amount equivalent to 15–20 mg/day of exogenous hydrocortisone. The recommended replacement dose for the treatment of GC-induced AI is therefore 15–20 mg of the short-acting GC. It may be taken in two (in the morning and at dinner time) or three doses per day [2]. Half to two-thirds of the daily dose should be administered in the morning to mimic the circadian rhythm of cortisol and avoid suppressing the morning ACTH secretion. An individualized approach concerning the patient’s symptoms and signs is recommended. Mineralocorticoid replacement is not needed in GC-induced AI.

### Prevention of Adrenal Crisis

During conditions that require an increased cortisol secretion, such as illness, patients with GC-induced AI should increase the GC dose to prevent the occurrence of adrenal crisis. In case of persistent vomiting, parenteral GC should be administered. It is important to ensure that the patient and/or the family are informed that during GC treatment the patient may require an administration of stress dose GC [66]. This concerns patients on GC treatment with a diagnosis of GC-induced AI, as well as patients with a probable risk of the HPA axis suppression (Table 2).

Peri-operative steroid cover in patients receiving supraphysiological doses of GCs should always be considered if there is a possibility that the patient may be adrenally suppressed. Recent guidelines from the Association of Anaesthetists, the Royal College of Physicians and the Society for Endocrinology UK [67] suggest administration of hydrocortisone 100 mg IV at induction, followed by a continuous infusion of hydrocortisone (200 mg over 24 h) for major surgery in case of patients receiving GCs in supraphysiological doses and those with GC-induced AI. Continuous IV hydrocortisone infusion should be preferred to intermittent bolus administration in the prevention and treatment of adrenal crisis during major stress [68]. Dexamethasone 6–8 mg IV may be used as an alternative, sufficient for a period of 24 h. Moderate procedures require 50 to 75 mg IV hydrocortisone (or equivalent) on the day of surgery and the first postoperative day. For minor surgical stress, the supplementation of hydrocortisone 25 mg IV (or equivalent) has been advised [69].

## 6. Conclusions

As much as 1–3% of the population is currently on chronic GC treatment, which may cause GC-induced AI. The predictors of this condition have not yet been well identified. GC-induced AI is an underestimated clinical condition, resulting in the risk of adrenal crisis while not properly treated. The diagnosis and treatment of GC-induced AI remain challenging. Several concerns are still raised regarding the right choice of the stimulation test to confirm the diagnosis. Patients with GC-induced AI require careful, prolonged endocrinological care, as the HPA axis recovery time cannot be predicted. The current literature lacks specific guidelines concerning the duration and regimen of GC withdrawal. Discontinuation of chronic GC therapy requires an individual approach, taking into consideration the risk of the HPA axis suppression in each treated patient.

## Figures and Tables

**Table 1:** Characteristics of glucocorticoid preparations.

Glucocorticoids	Equivalent Physiological Doses [mg/Day]	Duration of Action [Hours]	Mineralocorticoid Activity Relative to Hydrocortisone
Hydrocortisone	20	8–12	1
Prednisone	5	12–36	0.8
Methylprednisolone	4	12–36	0.5
Triamcinolone	4	12–36	0
Dexamethasone	0.75	36–72	0
Betamethasone	0.6	36–72	0

Data from: Nieman, L.K. Pharmacologic use of glucocorticoids. UpToDate. Accessed on 29 March 2021.

**Table 2:** Estimated risk of the suppression of the HPA axis with glucocorticoid therapy.

	Probable	Intermediate/Uncertain	Improbable
Treatment with prednisone (or equivalent)	≥20 mg/day for >3 weeks or ≥5 mg/day in the evening for more than a few weeks or Cushingoid appearance	10 to 20 mg/day for >3 weeks or <10 mg/day for more than a few weeks	< 3 weeks or alternate-day approach at a dose <10 mg
	Suggested approach		
	Gradual GC withdrawal	Gradual GC withdrawal	Cessation of GC therapy without previous gradual withdrawal is acceptable

GC, glucocorticoid. Data from: Furst, D. E.; Saag, K. G. Glucocorticoid withdrawal. UpToDate. Accessed on 29 March 2021.
